# Combined study of the ground and excited states in the transformation of nanodiamonds into carbon onions by electron energy-loss spectroscopy

**DOI:** 10.1038/s41598-019-40529-2

**Published:** 2019-03-07

**Authors:** Zhenbao Feng, Yangming Lin, Cunwei Tian, Haiquan Hu, Dangsheng Su

**Affiliations:** 1 School of Physical Science and Information Technology, Shandong Key Laboratory of Optical Communication Science and Technology, Liaocheng University, 252059 Liaocheng, China; 2 Max-Planck Institute for Chemical Energy Conversion, 45470 Mülheim, Germany; 3 Dalian National Laboratory for Clean Energy, Dalian Institute of Chemical Physics, Chinese Academy of Sciences, 116023 Dalian, China

## Abstract

The electron momentum density and sp^2^/sp^3^ ratio of carbon materials in the thermal transformation of detonation nanodiamonds (ND) into carbon nano-onions are systematically studied by electron energy-loss spectroscopy (EELS). Electron energy-loss near-edge structures of the carbon K-ionization in the electron energy-loss spectroscopy are measured to determine the sp^2^ content of the ND-derived samples. We use the method developed by Titantah and Lamoen, which is based on the ability to isolate the π^*^ spectrum and has been shown to give reliable and accurate results. Compton profiles (CPs) of the ND-derived carbon materials are obtained by performing EELS on the electron Compton scattering region. The amplitude of the CPs at zero momentum increases with increasing annealing temperature above 500 °C. The dramatic changes occur in the temperature range of 900–1300 °C, which indicates that the graphitization process mainly occurs in this annealing temperature region. Our results complement the previous work on the thermal transformation of ND-derived carbon onions and provide deeper insight into the evolution of the electronic properties in the graphitization process.

## Introduction

Nanodiamonds (NDs) and carbon nano-onions (CNOs), currently attract enormous attention and exhibit promising perspectives in applications. Nanodiamonds are excellent candidates as metal-free catalysts, drug delivery vectors and biomedical markers^1–4^. Potential applications of carbon onions include solid lubrication, anode materials for Li-ion batteries, electromagnetic shielding, metal-free catalysts, fuel cells and electrochemical energy storage^1,5–7^. At present, annealing nanodiamonds in an inert atmosphere or in vacuum is the most effective way to produce carbon onions^5,8^. The phase transform process includes nanodiamonds, bucky-diamond and carbon onions, which correspond to the sp^3^-bonded, mixed sp^2^/sp^3^-bonded and sp^2^-bonded carbon atoms, respectively. In the course of this transformation, the carbon hybridization changes from sp^3^ to sp^2^ and occurs from the surface inward. Raman spectroscopy, high-resolution transmission electron microscopy (HRTEM) and X-ray photoelectron spectroscopy (XPS) have been used to study this transformation under successive annealing treatments^8–10^. It is found that (i) annealing of nanodiamonds in an inert environment leads to the sp^3^-to-sp^2^ conversion beginning at the surface of smaller nanodiamonds at temperatures of 600–700 °C; (ii) annealing temperatures above 1000 °C, the majority of detonation nanodiamonds are partially or fully converted to carbon onions depending on the crystal size; (iii) an almost total completion of the transformation after annealing occurs at temperatures greater than 1500 °C. However, most of these studies have focused on the morphological and structural changes of annealing nanodiamonds during successive annealing temperatures. Until now, the full electronic structures of annealing nanodiamonds during the graphitization process of this sp^3^-to-sp^2^ conversion remains poorly understood. The numerous potential applications of nanodiamonds and carbon onions make it both scientifically interesting and technologically important to study the electronic properties during the phase transformation.

With the popularization of transmission electron microscopy (TEM) in laboratories, electron energy-loss spectroscopy (EELS) has become a standard analytical technique to determine the chemical composition, bonding information, optical properties and electronic structure of solids^11–13^. The low-loss region of EELS reflects the dielectric response of the material. The most commonly-used region of EELS, termed the electron energy-loss near-edge structure (ELNES), reflects the unoccupied density of states as well as the transition-matrix element^14^. Therefore, the information extracted from this region relates to the excited state electronic structure, bonding as well as orbital hybridization of the specimen. The Compton profile can be extracted from EELS in the large energy-loss region (more than 1000 eV), known as the electron Compton scattering from solids (ECOSS)^15^. In terms of electron microscopy, ECOSS is electron energy-loss spectrum (EELS) recording in diffraction mode at large scattering angle, performing an energy scan across the Bethe ridge^16–18^. ECOSS is a powerful tool to study the ground-state electronic momentum density of solids, especially for nanomaterials^17,19^. Recently, ECOSS measurements can be achieved in 1 minute for one available Compton profile^20–22^. The short recording time makes it practicable to study the Compton profile as a function of temperature, composition or orientation of the material^23^.

EELS has been used to study nanodiamonds and carbon onions^10,24^, where the spectra show typical diamond-like (sp^3^) and graphite-like (sp^2^) energy-loss near-edge structures, respectively. We have performed an initial study of the sp^2^ content with increasing temperature through measuring ELNES of annealing nanodiamonds based on the three-Gaussian (TG) method^24^. For the TG method, a small change of the energy upper limit for the fitting or the location of the second peak leads to a significant fluctuation of the sp^2^ fractions^25,26^. There is still much uncertainty of the sp^2^ fractions determined by the TG method for the nanodiamonds-to-carbon onions conversion.

In this work, we employ electron energy-loss spectroscopy to monitor both the sp^2^-bonded carbon fractions and the ground-state electronic momentum density distributions in the transformation of nanodiamonds into carbon onions with increasing the annealing temperatures. Through recording ELNES of ND-derived samples in the thermal transformation, the sp^2^ percentages were determined by the method developed by Titantah and Lamoen, which has been shown to give relatively accurate results^25^. A series of electron energy-loss spectra of samples in the transformation were measured in the Compton scattering regions. Valence Compton profiles of each carbon material in the transformation of nanodiamonds into carbon onions were obtained by ECOSS analysis.

## Results and Discussion

### Sp^2^/sp^3^ ratio

**Figure 1:**
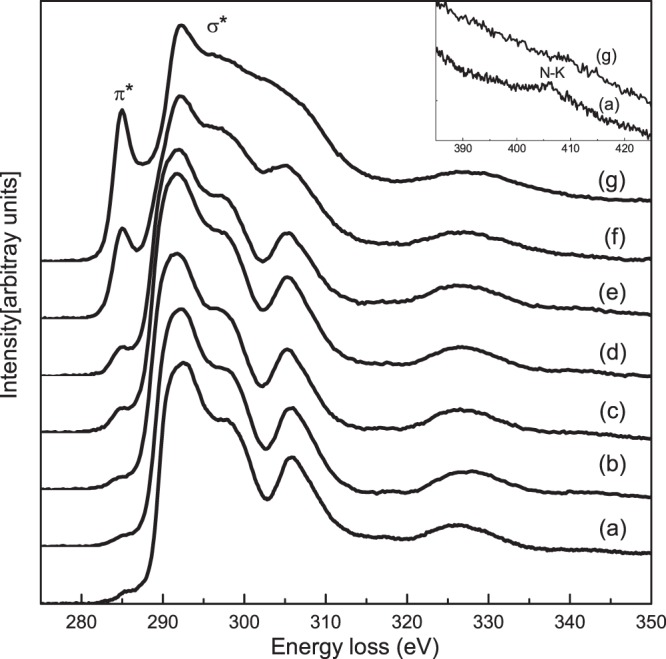
Experimental carbon K-edge fine structure (ELNES) spectra of (**a**) ND, (**b**) ND500, (**c**) ND700, (**d**) ND900, (**e**) ND1100, (**f**) ND1300 and (**g**) ND1500. The inset shows the region of nitrogen K-edge.

Figure 1 shows ELNES of the C-K edge from a series of ND-derived samples at different annealing temperatures after a power-law type background subtraction. We focused on two main typical peaks for the carbon materials in EELS, π^*^ and σ^*^ peaks. The π^*^ peak was located at approximately 285 eV, which was associated to the excitations of the 1 s core level electrons to unoccupied anti-bonding π^*^ states for sp^2^-hybridized carbon atoms. The sharp peak σ^*^ at approximately 292 eV arose from transitions of the 1 s core level electrons to unoccupied anti-bonding σ^*^ states. These two main features were observed in Fig. 1 for all spectra. With increasing temperatures, the relative intensity of the π^*^ peak increased. The rate of increase was negligible before the annealing temperature of 900 °C. This indicated the sp^2^-hybridized carbon atoms didn’t undergo pronounced increase before the annealing temperature of 900 °C. This was consistent with the onset of graphitization for ND when approaching 800–900 °C^8,27^. As shown in the inset, a clear signal for the nitrogen K-edge was present at 406 eV for the ND sample. Nitrogen is commonly found in detonation NDs synthesized with nitrogen-containing explosives^2,5^. The peak of the nitrogen K-edge disappeared for the ND1500 sample (carbon onions). The nitrogen dopants were lost in the process of reconstructive phase transformation from sp^3^-hybridized diamond to sp^2^-hybridized carbon onions^6,27^. No oxygen signal (K-edge of oxygen at 532 eV) was detected in these investigated samples.

The transformation of nanodiamonds into carbon onions involves sp^3^-bonded carbon atoms becoming sp^2^-bonded carbon atoms. The intermediate phase of carbon, named “bucky diamond”, with a nanodiamond core is covered by an onion-like carbon outer shell. Bucky diamond is formed by the mixed sp^2^/sp^3^ bonding of carbon atoms. The optical, electrical, mechanical and thermal properties of carbon materials are known to be particularly relevant to the bonding states of carbon atoms in a material. The sp^2^/sp^3^ ratio of carbon materials can be determined by several techniques, such as X-ray photoelectron spectroscopy, Raman spectroscopy, X-ray absorption near-edge spectroscopy and electron energy-loss near-edge spectroscopy^24,28,29^. Among them, electron energy-loss near-edge structure in combination with transmission electron microscopy is now the most powerful technique to quantify the ratio of sp^2^/sp^3^-hybridized carbons. The intensity of *I*_π*_ only stems from the contributions of carbons with sp^2^-hybridization. Both sp^3^- and sp^2^-hybridized carbons show strong contributions to the intensity of *I*_σ*_. The appearance of the π^*^ peak can be used as a sign of sp^2^-hybridized carbons. Thus, the hybridization ratio of the studied samples can be determined by applying Eq. (1)

1 $$s{p}^{2}=\frac{{[{I}_{{\pi }^{\ast }}/{I}_{total}]}_{sample}}{{[{I}_{{\pi }^{\ast }}/{I}_{total}]}_{100 \% s{p}^{2}reference,HOPG}}$$

**Figure 2:**
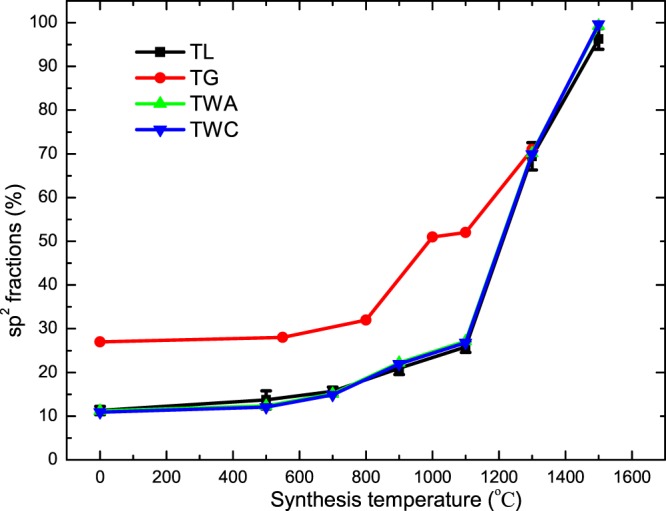
Sp^2^ fractions of ND-derived samples in the thermal transformation of nanodiamonds to carbon onions by TL method (black) along with results obtained from the TG (red)^24^, TWA (green) and TWC (blue) methods. The error bars (black) are plotted for our TL results.

The ratio is zero for purely sp^3^-hybridized carbons (diamond) and 100% for purely sp^2^-hybridized carbons (highly ordered pyrolytic graphite, HOPG). To avoid the possible influence of anisotropy on the samples, all ELNES measurements should be carried out at the magic-angle condition. Several approaches have been suggested and have discussed how to determine *I*_π*_ and *I*_*total*_, for example, “two-window method” with windows aligned on the edge onset (TWA), “two-window method” with windows centrally positioned to the major peaks (TWC), three-Gaussian (TG) methods and other model fitting methods^25,26,29^. Bernier *et al*. have reviewed these methods and discussed the advantages and disadvantages for each of them^26^. TWA, TWC and TG methods ignore the contributions in σ^*^ region for the *I*_π*_ calculation. In this work, we applied the Titantah and Lamoen (TL) method to determine the fraction of sp^2^-hybridized carbons during transformation from ND to CNO. This sp^3^/sp^2^ characterization technique is based on the ability to isolate the π^*^ features, independent of the position of the π^*^ and σ^*^ peak, the size of the energy window and the fewest fitting parameters. Figure 2 shows our calculated sp^2^ fractions using TL along with TG methods^24^. The TG method gives higher sp^2^ fractions during the transformation, especially at a low annealing temperature. Below 900 °C, the TG method showed that the sp^2^ fractions approached 30%, while no more than 20% was obtained for the TL method. Numerous studies have shown that sp^3^ to sp^2^ conversion mainly occurs on the surface of ND powders without altering the diamond core below 900 °C in an inert atmosphere. The sp^2^ fractions are attributed to a thin layer of amorphous and disordered carbon on the surface of ND or the surface reconstructs into graphitization at low annealing temperature. It can be noted that the sp^2^ fraction dramatically increased above 1100 °C for the TL results. This is consistent with results from Raman spectroscopy and TEM analysis ^8^. For comparison, we also calculated the sp^2^ fractions by TWA and TWC methods. The results are consistent with that of TL method, as shown in Fig. 2.

### Electron momentum density

**Figure 3:**
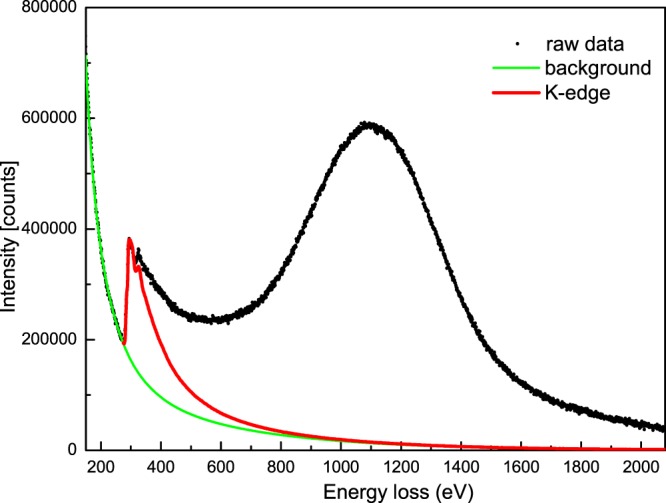
Raw EELS data of ND1500 (carbon onions) for ECOSS analysis along with the simulated background by parameterized elastic-inelastic scattering events and the contributions of the carbon K-ionization edge.

**Figure 4:**
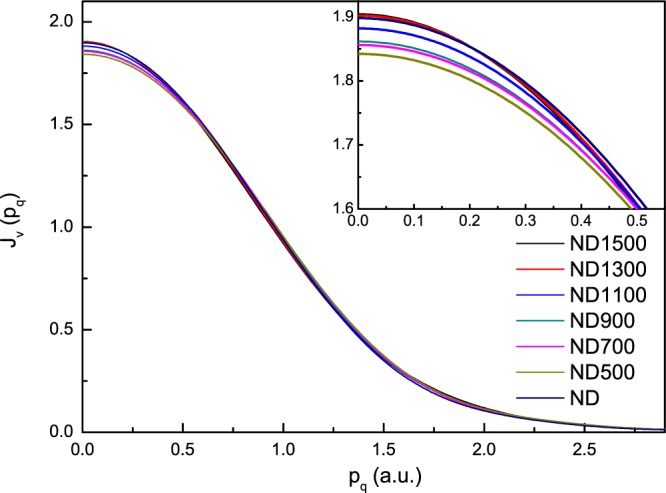
Valence Compton profiles of ND, ND500, ND700, ND900, ND1100, ND1300 and ND1500 (CNOs).

As a representation, the raw ECOSS data of ND1500, recorded by electron energy-loss spectroscopy, are shown in Fig. 3. The background was determined by parameterizing simulations of the combined elastic and inelastic scattering. The total exposure time was 2 min for each spectrum. This short recording time provided us with an opportunity to systematically study the electron momentum distributions of the transformation from ND to CNOs with annealing temperature. Figure 4 shows valence Compton profiles (CP) of ND, ND500, ND700, ND900, ND1100, ND1300 and ND1500. Details of the experimental and analytical procedures have been presented previously^19,20^. The amplitude of CPs increased with an increase of the annealing temperature above 500 °C. The amplitude of CP for ND500 was smaller than that of ND at zero momentum. This indicated a higher localization of the electron density distribution for ND in momentum space or higher delocalization in real space. Using Raman spectroscopy, the thermal transformation of nanodiamonds to carbon onions has been shown to start at 600 °C upon annealing in an argon atmosphere. Chemically, the raw detonation ND consists of 80–90% of the carbon in the mass, a few mass% of oxygen, hydrogen, nitrogen, and minor amounts of other impurities^5^. Oxygen and hydrogen emerge in the form of surface functional groups. The transformation is a multistep process. In general, the transformation starts with desorption of water and detachment of oxygen-containing surface functional groups on the surface of ND when heated up to approximately 200 °C. Increasing the temperature will remove functional groups like carboxyl, anhydride and lactone groups. Further increasing the temperature leads to the sp^3^-to-sp^2^ conversion from the surface inward. The functional groups and disordered carbon on the surface of ND are associated with the greater delocalization of the ground state charge density in real space.

**Figure 5:**
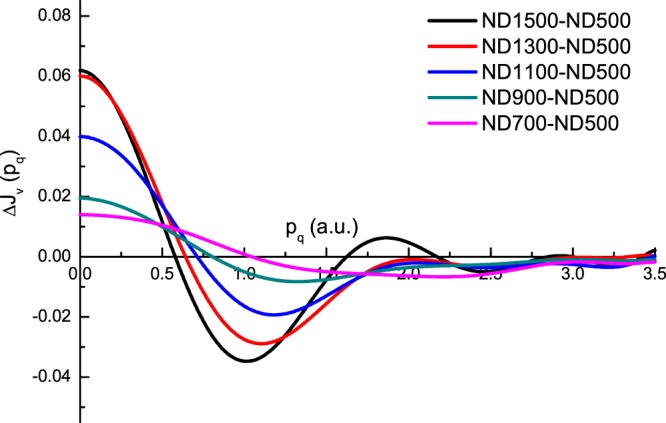
Profiles of ND700, ND900, ND1100, ND1300 and ND1500 with respect to ND500.

The differences between the profiles of ND700, ND900, ND1100, ND1300 and ND1500 with respect to ND500 are shown in Fig. 5. The evolution process of CPs for the transformation of “pure” sp^3^-hydridized diamond to sp^2^-hydridized carbon onions is clearly shown. The amplitude of CPs increased with respect to that of ND500. At p_q_ = 0, the ND1500 (carbon onions) profile was higher by 3% than that of ND500. This indicated a greater delocalization of the valence electrons in real space in the carbon onions. It should be mentioned that the density of nanodiamonds (3.3 g/cm^3^) was much higher than that of carbon onions (~1.9 g/cm^3^). The decrease of density in the transformation from nanodiamonds to carbon onions induces an increase in the particle volume. The volume expansion is expected to generate a larger delocalization of the charge density in carbon onions. In addition, we found the dramatic changes of CPs occur in the range of 900–1300 °C. We thus conclude that the majority of the sp^3^-to-sp^2^ conversion occurs in this temperature region. This conclusion is consistent with our ELNES study and previous work using other techniques^8,27^.

## Conclusions

Both the unoccupied and ground-state electronic properties of ND-derived carbon materials in the thermal transformation from ND to CNOs have been systematically studied by electron energy-loss spectroscopy using a transmission electron microscope. Electron energy-loss near-edge spectra of the carbon K-ionization were recorded at the optical axis to study the evolution of unoccupied states in the transformation. The sp^2^ fractions were determined by the TL method, which has been shown to give reliable and accurate results. Compared with the TG method, the TL method gives a lower sp^2^ content for ND-derived carbon materials at an annealing temperature below 1100 °C. Electron energy-loss spectra of a series of ND-derived carbon materials were recorded at large scattering angles (around 73 mrad) with short exposure time. The valence Compton profiles in the transformation were obtained by ECOSS analysis. Compared with “pure” NDs, the CPs of carbon onions indicated a substantially greater delocalization of the ground-state charge density. Investigation of both sp^2^ fractions and Compton profiles indicated the majority of sp^3^-to-sp^2^ conversion occurred at the thermal annealing temperatures of 900–1300 °C. This conclusion is consistent with previous works that used other techniques. ECOSS is shown to be a sensitive probe of the binding structure in nanomaterials. The short recording time enables the study of Compton profiles as a function of temperature. The ECOSS technique will be a powerful tool to study the ground-state electron momentum density distribution of a system and is highly sensitive to the change of ground-state wave functions, especially for carbon nanomaterials.

## Methods

### Electron energy-loss near-edge structure

The quantity measured in an EELS experiment is the double differential scattering cross section (DDSCS). In the first-order Born approximation, the DDSCS for fast electrons is given by^16^.

2 $$\frac{{\partial }^{2}\sigma }{\partial E\partial {\rm{\Omega }}}=\frac{4{\gamma }^{2}}{{a}_{0}^{2}{q}^{4}}\frac{{k}_{1}}{{k}_{0}}S({\bf{q}},E)$$

here *a*_0_ is the Bohr radius, *γ* is the relativistic factor, and *k*, *k*_0_ are wavevectors of the fast electron before and after scattering. $\hslash {\bf{q}}=\hslash ({{\bf{k}}}_{{\bf{0}}}-{{\bf{k}}}_{{\bf{1}}})$ is the momentum transfer in the scattering process. The dynamic structure factor $S({\bf{q}},\,E)$, a function of the momentum transfer **q** and energy loss *E*, is given by

3 $$S({\bf{q}},E)={\sum _{i,f}|\langle i|{e}^{i{\bf{q}}{\bf{r}}}|f\rangle |}^{2}\delta (E+{E}_{i}-{E}_{f})$$

here $|f\rangle$ and $|i\rangle$ are the final and initial states of the atoms, with energies *E*_*f*_ and *E*_*i*_, respectively, and r is the position operator. The sum is over all occupied initial and unoccupied final states.

The physical information of the DDSCS depends on the regime of the momentum transfer q and energy loss *E* in the dynamic structure factor [DFF]. To explain the near-edge structure in the EELS spectrum, the DFF following from band structure calculations can be rewritten as:

4 $$S({\bf{q}},E)={|\int {\psi }_{i}{e}^{i{\bf{q}}\cdot {\bf{r}}}{\psi }_{f}^{\ast }{d}^{3}{\bf{r}}|}^{2}\rho (E)$$

In Eq. (4), ${\psi }_{i}$ and ${\psi }_{f}$ are the initial and final state wave functions, respectively. $\rho (E)$ is the density of the final (or unoccupied) states (DOS). In this expression, the DFF is divided into two parts: the transition-matrix elements and the DOS term *p*(*E*). The transition-matrix term determines the basic edge shapes. For small momentum transfers $({\bf{q}}\cdot {\rm{r}}\ll 1)$, the operator reduces to a dipole approximation. The DOS term gives the fine structure on the basic near edge. Usually, the ELNES are recorded with the collection aperture centered on the optic axis in the TEM, and therefore, simply reflects the unoccupied density of states.

### Electron Compton scattering from solids

Most of the EELS experiments are performed around the unscattered electrons. If we tilt the incident beam through a few degrees so that only large-angled scattering electrons are recorded, a broad peak emerges at the high energy loss region, which is known as the electron Compton scattering spectrum. If the energy transfer is much greater than the binding energy of the atomic electron and the momentum transfer in the scattering process is significantly large relative to the inverse electronic orbital size, the binary-encounter impulse approximation (IA) is valid. Under the IA condition, the DFF can be given as^30^:

5 $$S({\bf{q}},E)=\frac{m}{\hslash q}J({p}_{q})$$

Here, *p*_*q*_ is the projection of the electron momentum along the direction of momentum transfer *q* and *J*(*pq*) is taken as the definition of the Compton profile

6 $$J({p}_{q})=2\pi {{\int }_{{p}_{q}}^{\infty }|\chi ({p}_{i})|}^{2}{p}_{i}d{p}_{i}$$

where $\chi ({p}_{i})$ is the momentum wave function of the initial state. Therefore, Compton profiles provide a direct experimental method to measure the ground state wave function of an electron in real space through the Fourier transform. The energy loss *E* in the IA condition is given by *p*_*q*_ as^17,31^

7 $$E={E}_{\max }+{p}_{q}{(2{E}_{\max })}^{1/2}$$

where *E*_max_ is the energy loss of Compton peak (Eq. (7) is obtained when ${m}_{e}=c=\hslash =1$). Eq. (7) is a critical step in the ECOSS analysis since it is used to convert the energy scale of a measured energy-loss spectrum to the momentum scale.

### Experimental

Purified ultra-dispersed nanodiamonds (UDD) were purchased from Beijing Grish Hitech Co. (China), which were produced by detonation and purified by acid washing. The average size of UDD was ~5 nm. Noncombustible contaminations were determined by an inductively coupled plasma optical emission spectrometer, and included Al < 50 ppm, Cu < 10 ppm, Cr < 10 ppm, Ca < 50 ppm, Fe < 50 ppm, Mg < 10 ppm and Ti < 10 ppm. Samples ND500, ND700, ND900, ND1100, ND1300 and ND1500 (CNOs) were produced by annealing purified UDD at 500, 700, 900, 1100, 1300 and 1500 °C for 4 h in an argon atmosphere, respectively. Each TEM specimen was prepared by ultrasonic vibration in pure ethanol for 10 mins and then depositing a drop of the suspension onto a TEM holey carbon grid. We have done EELS studied on specimens suspended over holes in the carbon support grid. Experiments were carried out on a FEI Tecnai G2 F20 microscope equipped with a Gatan GIF 963 spectrometer at an accelerating voltage of 200 kV. ELNES of each sample was recorded at the magic angle condition (collection angle ~1.5 mrad) with the entrance aperture centered on the optic axis under the parallel incident beam^32,33^. The energy resolutions were 0.8 eV (the full width at half maximum of the zero-loss peak, FWHM). For ECOSS acquisitions, thin regions with a diameter of approximately 800 nm were chosen by the convergent incident beam. The convergence angle was 3.5 mrad and the collection angle was 2.44 mrad. EELS spectra for ECOSS in the range of more than 2000 eV were recorded at a scattering angle of approximately 73 mrad. The Compton peak was located at approximately 1100 eV for each ECOSS measurements. The energy resolutions were 3 eV (FWHM) and the corresponding momentum resolution was 0.012 a.u. for the Compton profiles. Considering the recording conditions (beam convergence and collection angle), the total momentum resolutions were estimated to be in the order of 0.1 a.u.^17^. It is only possible to obtain such a good momentum resolution for photon Compton scattering by using a synchrotron radiation source. More than five measurements were taken at different positions for each sample to reduce the statistical error. The thickness or ELNES for each sample were measured before and after ECOSS measurements to make sure the sample had not suffered serious radiation damage.

## Supplementary information

Supplementary Information
