# Supplementary material for: Combined study of the ground and excited states in the transformation of nanodiamonds into carbon onions by electron energy-loss spectroscopy
Source: Sci Rep. 2019 Mar 7;9:3784. doi: 10.1038/s41598-019-40529-2 (PMC6405772; doi:10.1038/s41598-019-40529-2)
Supplement: Supplementary file 1 — Supplementary Information [file 41598_2019_40529_MOESM1_ESM.pdf]

SUPPLEMENTARY INFORMATION

Combined study of the ground and excited states in the transformation of nanodiamonds into carbon onions by electron energy-loss spectroscopy

Zhenbao Feng[1], Yangming Lin[2], Cunwei Tian[1], Haiquan Hu[1]&Dangsheng Su[3]

[1] School of Physical Science and Information Technology, Shandong Key Laboratory of Optical Communication Science and Technology, Liaocheng University, 252059, Liaocheng, China. [2] Max-Planck Institute for Chemical Energy Conversion, 45470 Mülheim, Germany. [3] Dalian National Laboratory for Clean Energy, Dalian Institute of Chemical Physics, Chinese Academy of Sciences, 116023, Dalian, China. Correspondence and requests for materials should be addressed to Z.F. (email:fengzhenbao@lcu.edu.cn)

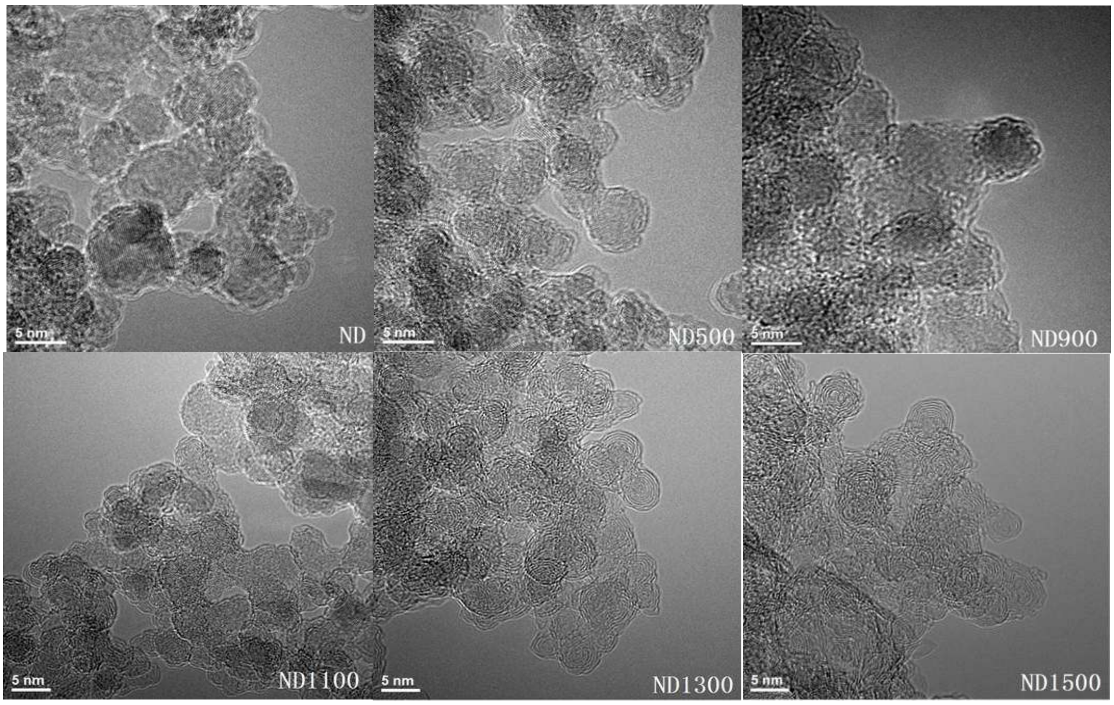

Supplementary Fig. S1. HRTEM images of ND, ND500, ND900, ND1100, ND1300 and ND1500.

In order to avoid anisotropy effects of the sample, all ELNES measurements were obtained at magic angle condition. This experimental condition was achieved by a highly oriented pyrolytic graphite crystal (HOPG) where no significant changes were found in spectra when tilting the sample out of the c-axis as shown in Figure 2.

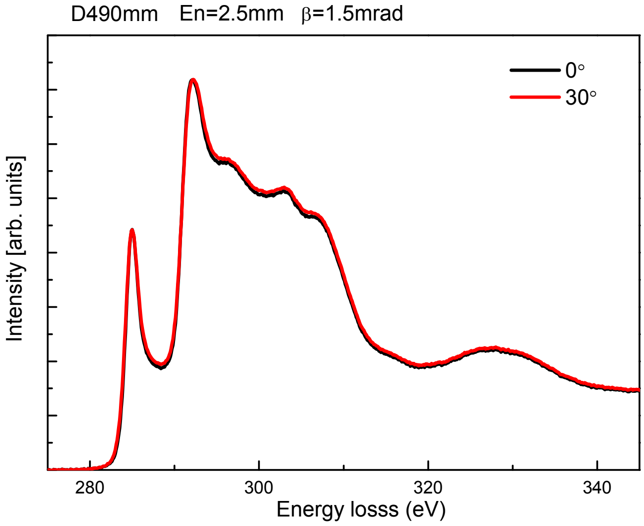

Supplementary Fig. S2. Energy loss spectra for the highly ordered pyrolytic graphite (HOPG) in different orientations ($\gamma = 0°$ and $30°$). The camera length were 490 mm and the spectrometer entrance aperture was set to 2.5 mm.

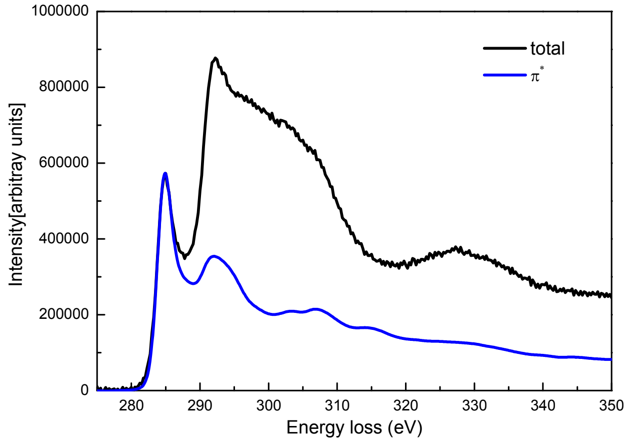

Supplementary Fig. S3. Experimental carbon K-edge fine structure spectrum of the ND1500 (black line) along with the fitting π^* spectrum (blue line). The π^* spectrum was determined by using the isolated π^* spectrum of graphite convolving with a Gaussian function[25]. The energy window of integral was chosen from edge onset up to 315 eV.
